# 2787. Carbapenem Versus Non-Carbapenem Based Therapy for Blood Stream Infection Caused by *Serratia Marcescen*s: A Multicenter Retrospective Cohort Study

**DOI:** 10.1093/ofid/ofad500.2398

**Published:** 2023-11-27

**Authors:** Abdallah Mughrabi, Julian Maamari, Timothy Philips, Afaq Alabbasi, Aislinn Brooks, Rinat Nuriev, Lisa Zenkin, Bertrand Jaber, Claudia Nader

**Affiliations:** St. Elizabeth's Medical Center - Boston University School of Medicine, Boston, Massachusetts; St. Elizabeth's Medical Center - Boston University School of Medicine, Boston, Massachusetts; St. Elizabeth's Medical Center - Boston University School of Medicine, Boston, Massachusetts; St. Elizabeth's Medical Center - Boston University School of Medicine, Boston, Massachusetts; Steward St. Elizabeth's Medical Center of Boston, Rutland, Massachusetts; St. Elizabeth's Medical Center - Boston University School of Medicine, Boston, Massachusetts; St. Elizabeth's Medical Center - Boston University School of Medicine, Boston, Massachusetts; St. Elizabeth's Medical Center - Tufts School of Medicine, Boston, Massachusetts; St. Elizabeth's Medical Center - Tufts School of Medicine, Boston, Massachusetts

## Abstract

**Background:**

Carbapenems were commonly prescribed for "SPACE" organisms. Recent IDSA guidance recommends narrower-spectrum beta-lactams for lower AmpC-induction risk bacteria, like Serratia. Prior studies focused on other Enterobacterales, with limited evidence on *Serratia* bacteremia. This study compares carbapenem-containing (CBCT) and non-carbapenem-containing active empiric/definitive therapy (NCBCT) for *Serratia* bacteremia.

**Methods:**

We retrospectively reviewed records of adults (age ≥18 years old) with *Serratia* bacteremia hospitalized in 7 hospitals in Massachusetts over 7 years (2015-2022). IRB approval was obtained. Baseline characteristics, treatment details and outcome variables were extracted from medical charts. Chi Square and Mann-Whitney U tests were computed using SPSS statistical package.

**Results:**

73 out of 128 patients were included in the study after applying exclusion criteria **(Figure 1).** Mean age was 58.8 years. CBCT was prescribed in 23.3%. No significant differences were observed in baseline characteristics, bacteremia sources or severity; however, CBCT patients had longer definitive therapy and more prolonged hospital stay **(Table 1)**. The 30-day mortality rate was not significantly different between both groups (14.8% NCBCT vs. 0% CBCT, P=0.102). While there was no difference in antimicrobial failure with a relative risk of 1.415 (95% CI 0.38-5.26, P=0.603), the infection-related hospital readmission rate was significantly higher in the CBCT group.

Study exclusion flow-chart.

**Figure:**
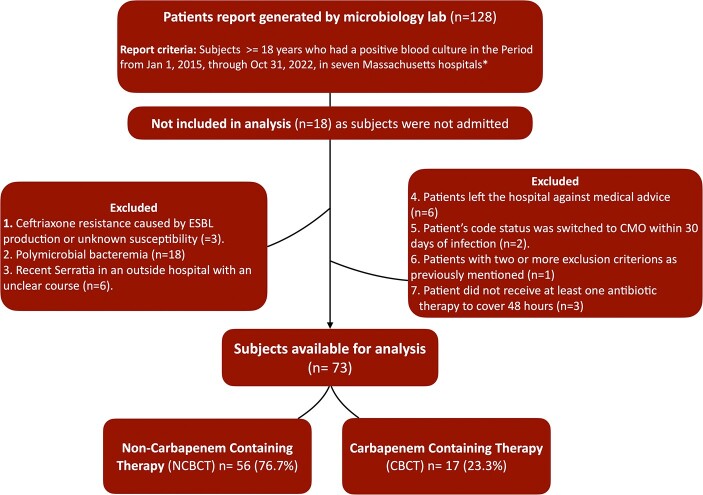

ESBL phenotypically defined by ceftriaxone resistance (MIC <1)

Characteristics and outcomes of patient with Serratia bacteremia treated with a carbapenem versus a non-carbapenem therapy regimen.

**Figure:**
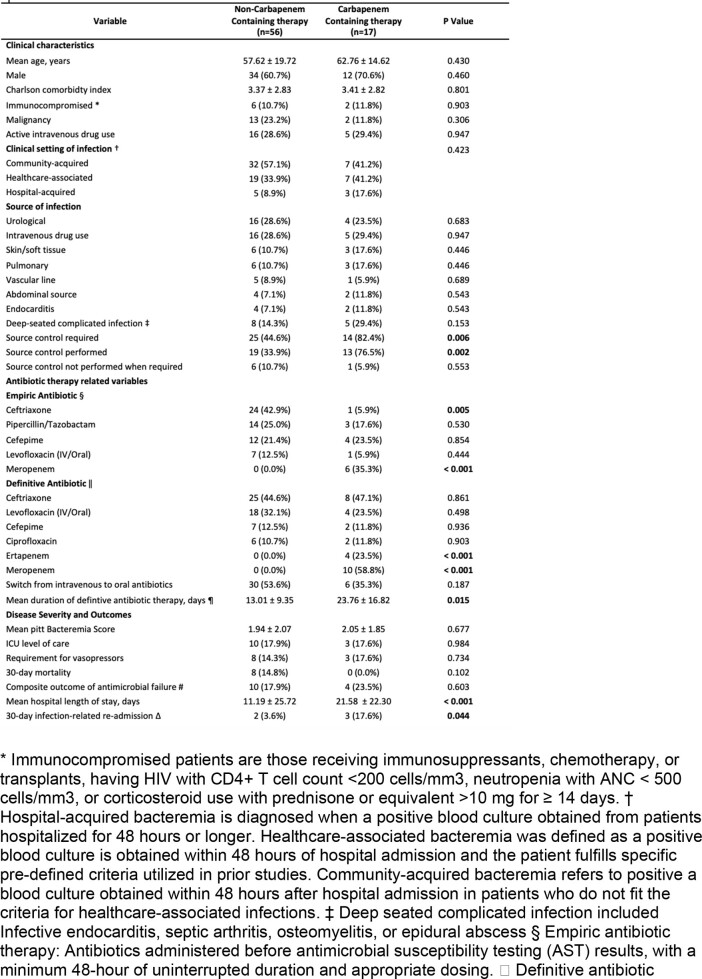

* Immunocompromised patients are those receiving immunosuppressants, chemotherapy, or transplants, having HIV with CD4+ T cell count <200 cells/mm3, neutropenia with ANC < 500 cells/mm3, or corticosteroid use with prednisone or equivalent >10 mg for ≥ 14 days. † Hospital-acquired bacteremia is diagnosed when a positive blood culture obtained from patients hospitalized for 48 hours or longer. Healthcare-associated bacteremia was defined as a positive blood culture is obtained within 48 hours of hospital admission and the patient fulfills specific pre-defined criteria utilized in prior studies. Community-acquired bacteremia refers to positive a blood culture obtained within 48 hours after hospital admission in patients who do not fit the criteria for healthcare-associated infections. ‡ Deep seated complicated infection included Infective endocarditis, septic arthritis, osteomyelitis, or epidural abscess § Empiric antibiotic therapy: Antibiotics administered before antimicrobial susceptibility testing (AST) results, with a minimum 48-hour of uninterrupted duration and appropriate dosing. || Definitive antibiotic therapy (i.e., antibiotics administered after the AST results, also with a minimum of 48-hour duration). ¶ Duration of total definitive antibiotic therapy was counted from day of AST results until last day of definitive antimicrobial therapy. # Microbiological failure, microbiological relapse, in- hospital mortality, 30-day mortality, infection-related hospital readmission, or recurrent Serratia bateremia within 30 days. Microbiological failure refers to the growth of Serratia after 48 hours of definitive antibiotic therapy, while microbiological relapse is the regrowth of Serratia after a negative blood culture (Kunz Coyne et al., 2023). Note: Two patients had recurrence of Serratia bacteremia within 30 days, neither were resistant to third generation cephalosporins. Δ Infection-related re-admission was defined when a patient is readmitted not solely for a procedure or surgery, and they experience fever, worsening leukocytosis, or required escalation of antimicrobial therapy from the definitive therapy of the index admission.

**Conclusion:**

We observed no significant differences in antimicrobial failure or mortality between CBCT and NCBCT groups. Narrower beta-lactam therapy may be appropriate when possible. In fact, CBCT was associated with prolonged hospital length of stay, which may be due to challenges in switching to outpatient therapy and discharging to lower acuity care settings. CBCT was also associated with more infection-related readmissions, which could be attributed to patient characteristics (IVDU and deep-seated infections) and frequent dosing requirements of meropenem impacting adherence. Our retrospective study is limited by a small size and overlap in definitive therapy and further prospective studies are needed to confirm our findings.

**Disclosures:**

**All Authors**: No reported disclosures

